# Gilteritinib-associated hand-foot syndrome: a novel dermatologic reaction in refractory FLT3 Acute myeloid leukemia

**DOI:** 10.1016/j.lrr.2026.100585

**Published:** 2026-04-12

**Authors:** Jack T. Seki, Jad Sibai, Maria Agustina Perusini, Hassan Sibai

**Affiliations:** a Division of Medical Oncology and Hematology, Princess Margaret Cancer Centre, University Health Network, Toronto, Canada; b Leslie Dan Faculty of Pharmacy, University of Toronto, Toronto, Canada; c Department of Medicine, University of Toronto, Ontario, Canada

**Keywords:** Hand-foot syndrome, Second generation FLT3-TKIs, AML

## Abstract

Objective: Second generation tyrosine kinase inhibitors (TKIs) such as gilteritinib, characterized by minimal EGFR and absent VEGFR inhibition, are in theory associated with low dermatologic toxicity. This case report brings to the awareness that the opposite may occur and emphasize the need for attentive pharmacovigilance.

Methods: An elderly woman presented to us with relapsed/refractory (R/R) FLT3-ITD AML following azacytidine treatment, received single-agent TKI gilteritinib, selected for its greater potency and specificity. Unexpectedly, she developed a severe hand-foot skin lesion requiring treatment interruption.

Results: After receiving two cycles of gilteritinib 120mg orally daily without therapeutic response, the dose was escalated to 200mg in accordance with RCT guidelines. After one week, the patient developed dry skin and mild erythema of the hands and feet, which progressed to a severe hand-foot syndrome the following week.

Conclusion: This unprecedented adverse event reporting suggests that the FLT3-specific TKI gilteritinib can induce cutaneous toxicities, through dose-dependent inhibition of proangiogenic pathways.

## Highlights

• Unexpected toxicity: Gilteritinib, a second-generation FLT3-specific TKI with minimal EGFR/VEGFR inhibition, was presumed to have low dermatologic toxicity. This case challenges that assumption.
• Clinical presentation: Initial pruritus followed by desquamating rash on limbs, rapid progression to severe hand-foot syndrome requiring treatment interruption.
• Dose relationship: Adverse reactions emerged after dose escalation from 120 mg to 200 mg daily, suggesting dose-dependent inhibition of proangiogenic pathways as a mechanism.
• Novelty: First reported case of severe cutaneous toxicity associated with gilteritinib in this clinical context. This may be linked to off-target inhibition of proangiogenic pathways, such as VEGFR, PDGFR, RET and KIT.
• Underscores the need for rigorous pharmacovigilance even with agents thought to have low dermatologic risk.

## 1 Introduction

Mutations of FMS-like tyrosine kinase 3 (FLT3) are commonly occurring in about 30% of acute myeloid leukemia (AML), with internal tandem duplications (ITD) comprising the majority (⁓ 25%) of cases [1]. FLT3-ITD AML initially responds to traditional chemotherapy, but carries significantly higher relapse risk than non-FLT3-mutated AML variants. As FLT3 activation promotes leukemic proliferation via tyrosine kinase receptor overexpression, small-molecule FLT3 tyrosine kinase inhibitors (TKIs) have been developed for relapsed/refractory (R/R) FLT3-ITD AML cases [2].

TKI therapeutic integration has improved clinical outcomes of patients, though the toxicity profile of first-generation broad-spectrum TKIs are notable for cytopenia, transaminitis, nausea/vomiting and skin, heart, and kidney adverse events [3,4]. Gilteritinib, a second-generation TKI with greater potency and specificity, is approved as monotherapy for FLT3-ITD R/R AML and offers a more favorable toxicity profile, primarily diarrhea, anemia, transaminitis in comparison [5,6]. This mechanistic context frames our observation of a dose-dependent acral rash in a patient receiving gilteritinib.

To our knowledge, we are reporting the first cased of Palmar-Plantar Erythrodysesthesia Syndrome, also known as hand-foot syndrome (HFS) following single agent gilteritinib treatment for refractory FLT3-ITD AML, a side effect previously linked only to first-generation TKIs.

## 2 Case report

A 75-year-old female presented with pancytopenia and circulating blasts. Bone marrow (BM) biopsy and aspirate were consistent with AML. Her ECOG performance score was 2. Given no approval for venetoclax funding at the time, she was treated with azacytidine, which was well-tolerated but required multiple blood transfusions. 4 months following initial diagnosis, BM biopsy showed an increased blast count (80%). Cytogenetic tests showed 46XX with t(2;11) in 10 metaphases. Though undetected at diagnosis, follow-up molecular testing was positive for the FLT3-ITD mutant allele. Using next-generation sequencing, the FLT3 variant (p. Glu598_Tyr599ins16) was detected at 29%, along with a WT1 mutation of unknown significance.

Given the disease’s unresponsiveness to azacytidine and the presence of the FLT3-ITD mutation, the patient was started on a single-agent gilteritinib therapy. Initially, she received 120mg dose of orally administered gilteritinib per day. Her complete blood count (CBC) showed hemoglobin at 62g/dL, platelets at 72 × 10^9^/L and white blood cells at 18.9 × 10^9^/L. She experienced early tumour lysis syndrome, which was responsive to rasburicase, allopurinol and hydration. Throughout the course of treatment, she developed febrile neutropenia and was treated with antibiotics.

**Fig. 1:**
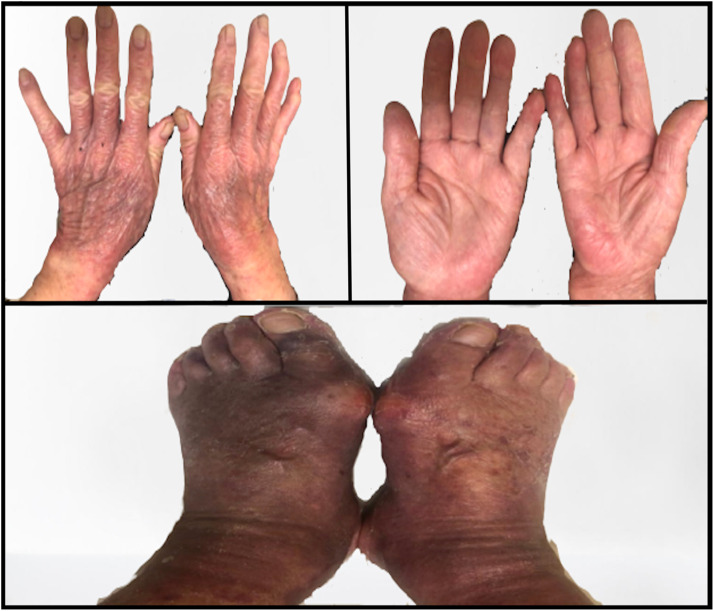
Images of hands (top-left; dorsal | top-right; palmar) and feet (bottom; dorsal), 1-week post-dosage escalation to 200m.

**Fig. 2:**
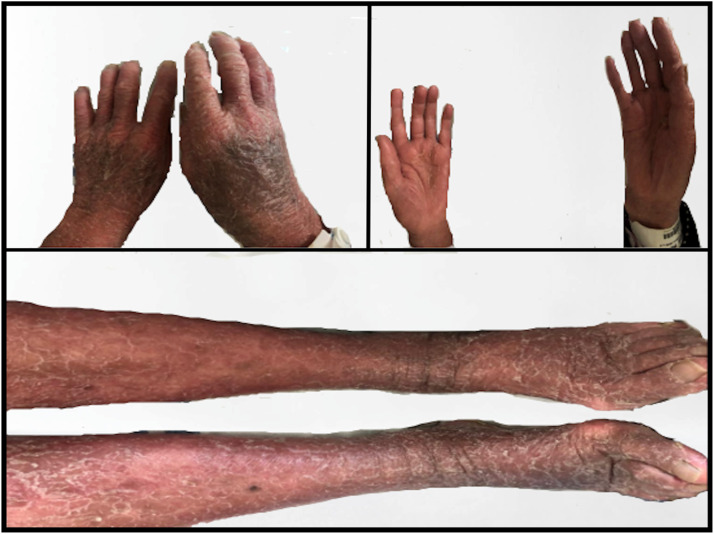
Images of hands (top-left; dorsal | top-right; palmar) and feet (bottom; dorsal including lower limbs), 26 days post-dosage escalation to 200mg.

After 2 cycles of 120mg gilteritinib daily**,** there was no improvement in cytopenia or the number of persistent circulating blasts. In keeping with the guidelines of the gilteritinib randomized control trial, the dosage was increased to 200mg [7]. Her CBC prior to dose escalation showed haemoglobin at 85g/dL, white blood cells at 1 × 10^9^/L and platelets at 38 × 10^9^/L. However, within one week of increasing the dosage, the patient started developing dry skin and mild erythema in her hands and feet. The following week, she returned with severe HFS (Grade 3 CTCAE v5 [8].), prompting us to withhold the drug (Fig. 1) and consulted dermatology. A skin biopsy was scheduled but did not complete due to thrombocytopenia. The patient developed a desquamating rash that initially appeared on her feet, before progressing to involve both her upper and lower limbs, with pronounced involvement in the palmar and dorsal aspects of the hands and feet (Fig. 2). Itchiness was only present in the first few days. Given temporal association and rash morphology, the complication is likely secondary to gilteritinib treatment. No potential drug-drug interactions was noted.

After withholding the drug for 4 weeks, the patient was re-challenged with gilteritinib. She first received 80mg without reoccurrence of HFS. After two weeks, Venetoclax was added to her treatment. Unfortunately, 2 months later, she suffered from a respiratory infection with underlying multiple bouts of febrile neutropenia and passed away without a response. Throughout this period, HFS did not recur.

## 3 Discussion

Hand-foot syndrome (HFS) is a dose-limiting toxicity commonly associated with first-generation TKIs such as sorafenib (10–62%) and sunitinib (10–28%)⁶. Although previously unlinked to gilteritinib, the observed dose-dependent, acral distribution is consistent with TKI-induced HFS. Gilteritinib is a multi-targeted TKI with high selectivity for FLT3 and AXL and minimal inhibition of EGFR or other kinases commonly implicated in dermatologic toxicity. This may explain the absence of EGFR-mediated rash, as seen with both gilteritinib [9] and quizartinib [10], opposite is true for midostaurin [11] and sorafenib [12] (Table 1). That said, inhibition of proangiogenic pathways such as VEGFR and PDGFR may impair vascular repair mechanisms and contribute to cutaneous toxicity [13]. TKIs with stronger EGFR inhibition typically produce more pronounced skin reactions, further supporting the low dermatologic toxicity observed with gilteritinib. Still, off-target inhibition of RET, KIT, or PDGFR at clinically relevant drug concentrations cannot be entirely ruled out and may plausibly contribute to the development of skin reactions [14].

**Table 1:** Dermatologic Toxicity Comparison: Gilteritinib vs Other TKIs.

**Drug**	**Target(s)**	**Common Skin Toxicities**	**Rash frequency**	**Mechanism of Skin Effects**	**Ref.#**
Gilteritinib	FLT3, AXL	Rare; no consistent rash or pruritus reported	No formalized documentation as a single agent, very rare	Minimal EGFR or lack VEGFR inhibition	9
Quizartinib	FLT3	Rash, dry skin (less common)	Grade 1-2:14%, Grade 3:<1%	Selective FLT3 inhibition, minimal dermatologic toxicity	10
Midostaurin	FLT3, KIT, PDGFR	Rash, dry skin, pruritus (mild)	Exfoliative Dermatitis (62%) (14% severe)	Multikinase inhibition (mild PPE-like dermatologic symptoms)	11
Sorafenib	FLT3, VEGFR, RAF	Rash, HFS, alopecia, erythema	Yes, high all grades: 76%, grade 3: 20%	VEGFR/RAF inhibition → keratinocyte stress. Classic HFSR with hyperkeratosis, erythema, and pain	12
Feline McDonough Sarcoma oncogene-like tyrosine kinase 3 (FLT3) AXL Receptor Tyrosine Kinase (TAM family) (AXL) Epidermal Growth Factor Receptor (EGFR) Vascular Endothelial Growth Factor Receptor (VEGFR) Platelet-Derived Growth Factor Receptor Stem Cell Factor Receptor (KIT) CD117 personal protective equipment (PPE) Hand foot skin reaction (HFSR)					

Iijima et al.’s study demonstrated a dose-dependent increase in sorafenib-induced HFS: 0% at 100mg, 20% at 200 mg, 50% at 400 mg, 86% at 800 mg [15]. Similar trends were observed in case reports describing the onset of HFS at higher sorafenib doses within a single patient (Sil et al) [16]. Our patient tolerated an initial gilteritinib dosage of 120mg without experiencing HFS, but developed symptoms at 200mg. Could an escalated dose of 200 mg daily represented a threshold dose sufficiently high, precipitating such an idiosyncratic devastating adverse event? Gilteritinib exhibits dose-proportional pharmacokinetics with extensive tissue distribution, and the observed dose-dependent onset and resolution of HFS in our patient suggest that higher daily doses may trigger off-target effects, reinforcing the dose-limiting nature of this adverse event consistent with TKI-induced HFS [13,17]. While the exact pathogenesis of TKI-induced HFS remains unclear, proposed mechanisms include impaired vascular and fibroblast repair [18], eccrine gland toxicity [19], and off-target kinase inhibition histologic changes have been described in patients receiving multitargeted TKIs such as sorafinib and sunitinib [13,20].

The patient presented with redness, swelling, desquamation, itchiness, and impaired ambulation. Dermatologic assessment findings consistent with drug-induced hand-foot syndrome (HFS). A biopsy of the rash and excision of a suspected basal cell carcinoma on the right cheek were recommended, but deferred due to thrombocytopenia. Though the most common clinical feature of HFS is characterized by a rash and/or swelling that is restricted to the palms and soles, TKI-induced clinical variants accounts for broader presentation, including scalp dysesthesia, angular cheilitis, perianal rashes, and facial erythema, underscoring ambiguity in its diagnostic criteria [21]. Coupled with an unknown pathogenesis, HFS remains a challenging task to delineate (Table 2).

**Table 2:** clearly separate early systemic effects (TLS) and (DS) from delayed dermatologic toxicity (HFS), reinforcing the novelty and specificity of the case.

**Feature**	**Tumor Lysis Syndrome (TLS)**	**Hand-Foot Syndrome (HFS)**	**Differentiation Syndrome (DS)**
**Presented by patient**	yes	yes	no
**Timing of Onset**	Early (within first week)	Delayed (week 10 of treatment)	Early (typically days 2–21)
**Systemic Features**	Electrolyte disturbances, renal risk	• Brief pruritus lasting a few days • Extensive xerosis • Erythematous morbilliform rash on upper and lower limbs • Most prominent on dorsum of hands and feet • Desquamation extending beyond ankles and wrists • Palms and soles spared • Nails and mucosa normal • No erosions or skin tenderness	Fever, dyspnea, hypotension, edema
**Rash Distribution**	None	• Rash started in feet, spread to hands • Small, non‑painful papule near right lateral canthus (occasional bleed) • Grew to ∼1 cm over 2 months of treatment • Nodule with central keratinous material and peripheral hemorrhage (infraorbital, near lateral canthus) • Surrounding skin normal • Nails and mucosa unaffected • Findings consistent with cumulative cutaneous toxicity	Generalized or facial; not acral
**Associated Drug Mechanism**	Rapid cytoreduction	Cumulative cutaneous toxicity	Leukemic cell maturation
**Drug Implicated**	Gilteritinib [9]	Gilteritinib [9]	Gilteritinib [9]
**Supportive Features in Case**	Hyperuricemia, neutropenia	Acral rash, delayed onset	Absent systemic inflammatory signs
**Interpretation**	Treatment-disease-related complications	Likely drug-induced HFS	Unlikely DS due to timing and features

Drug safety profiles continue to evolve over time through post-marketing surveillance, as real-world data may reveal adverse events not observed in clinical trials. While rash was not highlighted in the ADMIRAL trial [22], it is listed in the Canadian product monograph as a potential manifestation of Differentiation Syndrome (DS); however, the delayed onset and acral distribution observed in our case are more consistent with hand-foot syndrome (HFS). Our patient developed an acral rash with transient pruritus, beginning on the feet and spreading to the hands at week 10 of therapy. The delayed onset, extremity-predominant distribution, and absence of systemic inflammatory are more consistent with hand-foot syndrome (HFS) than classical DS. Nonetheless, the inclusion of rash in DS labeling invites consideration of atypical or overlapping presentations, particularly given gilteritinib’s multi-kinase inhibition profile, which may contribute to diverse cutaneous toxicities with expanded clinical use. Furthermore, tumor lysis syndrome (TLS) occurred early in the treatment course, consistent with high disease burden and rapid cytoreduction. In contrast, the acral rash emerged well beyond the typical window for DS, which usually presents within the first 2–3 weeks. This temporal separation reinforces the interpretation that the cutaneous findings represent a distinct, cumulative toxicity rather than a late manifestation of DS.

Despite this complication, gilteritinib remains an effective option for the treatment of refractory FLT3-ITD AML. It has proven significantly potent by withstanding mutations that confer resistance against alternative AML-oriented TKI options, namely, midostaurin and quizatinib [6]. Gilteritinib’s dose-escalation trials have also shown a higher response rate among patients with FLT3-ITD AML. Dose-escalation trials have shown improved response rates, suggesting that establishing a maximum tolerated dose (MTD), similar to sorafenib’s 400 mg, may help prevent HFS while preserving therapeutic efficacy.

Finally, it is important to note that FLT3 inhibitors, especially gilteritinib are increasingly visible and reported with significant cutaneous toxicity in the real-world practice. However, these skin adverse reactions appeared underrepresented by a recent population-based pharmacovigilance FAERS disproportionality analysis [23]. Expanding on the earlier publications of gilteritinib-associated dermatological reactions, two recent case reports further strengthened the spectrum of skin reactions data ranging from pyoderma gangrenosum, as the result of severe ulcerative neutrophilic dermatoses requiring drug discontinuation, to non-ulcerative inflammatory processes amenable to corticosteroid treatment [24,25]. Collectively, these studies highlighted the need to monitor potential cutaneous reactions in patients initiating FLT3 inhibitor therapy, and underscore the value of skin biopsy as a means to distinguish drug-related from disease-related cutaneous adverse events.

## 4 Conclusion

To the best of our knowledge, this is the first report of gilteritinib-associated HFS. The dose-dependent onset and resolution suggest a threshold effect, warranting further investigation into its cutaneous toxicity profile.

## Funding information

The authors received no specific funding for this work.

## Data availability statement

The data that support the findings of this study are available on request from the corresponding author. The data are not publicly available due to privacy or ethical restrictions.

## Ethics statement

Consent for the publication and academic discussion of the case was obtained from the patient.

## Patient consent statement

Informed consent was obtained.

## Informed consent

This is a case report; therefore, ethic approval was not required and patient consent was obtained

Hassan Sibai, MD

Associate Professor, Department of Medicine University of Toronto

Division of Medical Oncology and Hematology

Princess Margaret Cancer Centre

610 University Avenue, OPG 6th Floor – 6-718

Toronto, ON M5G 2M9

## CRediT authorship contribution statement

**Jack T. Seki:** Writing – review & editing, Writing – original draft. **Jad Sibai:** Writing – review & editing, Writing – original draft. **Maria Agustina Perusini:** Data curation. **Hassan Sibai:** Supervision, Resources, Project administration, Investigation, Data curation, Conceptualization.

## Declaration of competing interest

All other authors have no conflicts to report.
